# Trends and patterns of pharmacist-administered vaccination in a predominantly Western Australian pharmacy group: retrospective analysis of routinely collected data

**DOI:** 10.1007/s11096-026-02221-0

**Published:** 2026-09-15

**Authors:** Ashenafi Kibret Sendekie, Petra Czarniak, Tin Fei Sim, Anneika Tonkin, Kristian Ray, Leanne Chalmers

**Affiliations:** 1 Curtin School of Diagnostic and Therapeutic Sciences, Faculty of Health Sciences, Curtin University, Bentley, WA 6102, Australia; 2 enAble Institute, Curtin University, Bentley, WA 6102, Australia; 3 Pharmacy 777 Group Administration, Osborne Park, WA 6017, Australia

**Keywords:** Australia, Community pharmacy, Immunisation, Pharmacist, Rural, Vaccination

## Abstract

**Introduction:**

Pharmacists are integral to vaccination strategies globally and within Australia’s National Immunisation Program. However, limited evidence exists regarding how uptake of pharmacist-administered vaccination varies across demographic groups and geographic locations.

**Aim:**

This study aimed to analyse trends and patterns in pharmacist-administered vaccinations delivered within a predominantly Western Australian community pharmacy network.

**Method:**

A retrospective descriptive analysis was undertaken using de-identified routine vaccination records from January 2021 to April 2025. Data were sourced from a booking system (January 2021–March 2024) and a clinical record system (April 2024–April 2025) of a selected pharmacy group. Temporal trends and vaccination patterns, stratified by demographic (age and sex) and geographical distributions, including Modified Monash Model (MMM) category and proximity to medical centres, were assessed. Vaccination rates per 100, 000 population were calculated across age, sex, and MMM groups using Western Australian vaccination records as the numerator and corresponding population estimates as the denominator.

**Results:**

Over 1.1 million vaccination records spanning 19 vaccine types were analysed. COVID-19 vaccines accounted for 59.4% of all vaccinations, peaking between late 2021 and early 2022. Influenza vaccination showed consistent seasonal peaks (March–June), with a decline in 2024 compared with previous years. Administration of other routine vaccines was variable but gradually increased, particularly in 2024. Vaccination rate per 100,000 population was highly variable among age groups, with an overall lower vaccination rate among younger individuals (aged 5–19 years) and older people (aged ≥85 years). Vaccination services were concentrated in metropolitan areas and pharmacies near medical centres, with a similar pattern between males and females.

**Conclusion:**

Community pharmacists played a vital role in vaccination delivery, particularly during the COVID-19 pandemic and seasonal influenza periods. Routine vaccine administration has the potential to expand further with targeted health campaigns to improve public awareness and uptake across all populations and regions. Further research should develop strategies to enhance equitable access across geographic areas.

**Supplementary Information:**

The online version contains supplementary material available at https://doi.org/10.1007/s11096-026-02221-0.

## Impact statements

- The demonstrated substantial contribution of community pharmacies to vaccination services, particularly during periods of peak demand such as COVID-19 and seasonal influenza, reinforces their role as accessible immunisation providers.
- Findings such as lower vaccine uptake in community pharmacies among patients aged 5–19 years and younger and ≥85 years support data-driven service planning and workforce development and strengthen collaboration with other vaccinators to optimise vaccination delivery.
- Enhancing the roles of community pharmacists in the routine immunisation program has the potential to improve equitable access and increase vaccination uptake across diverse population groups and rural and remote areas.

## Introduction

Vaccination remains a cornerstone of public health, significantly reducing infectious diseases and associated morbidity and mortality worldwide [1]. Since the World Health Organization’s (WHO’s) Expanded Program on Immunisation (EPI) was established in 1974, global immunisation coverage has improved considerably, contributing to marked declines in vaccine-preventable diseases (VPDs), particularly among children [2]. Despite this progress, VPDs continue to cause avoidable illness and death, especially among vulnerable populations, including children, older adults, and socioeconomically disadvantaged groups [3]. Persistent challenges, including inequitable access to vaccination services [4], vaccine hesitancy and complacency [5, 6], and logistical barriers such as supply and distribution constraints [7] continue to limit optimal coverage. Addressing these challenges requires integrated life-course immunisation strategies embedded within accessible primary healthcare systems [8].

Pharmacists have increasingly been recognised as important contributors to vaccination delivery globally [9, 10]. In many high- and middle-income countries, pharmacist-administered vaccination has been incorporated into national immunisation strategies, supported by evidence demonstrating improvements in accessibility, convenience, and vaccine uptake [11–14]. Community pharmacies, characterised by their wide geographical distribution, extended opening hours, and walk-in availability, have become key access points for vaccination services [15–20].

In Australia, pharmacist-administered vaccinations have been progressively introduced and expanded across jurisdictions [21, 22]. Pharmacy-based vaccination was first introduced in 2014, initially for influenza vaccination in Queensland, and has since expanded nationally following regulatory and policy changes [23]. These reforms enabled appropriately trained pharmacists to deliver a broad range of vaccines across all jurisdictions, representing a significant evolution in both scope of practice and models of service delivery [24–26]. Between 2016 and 2021, the proportion of vaccinations administered by pharmacists rose from negligible levels (less than 0.001%) to approximately 2.7% [24], reaching 7.3% nationally by 2021 [27]. This role expanded further during the COVID-19 pandemic, with pharmacists serving as key access points and administering more than 12 million COVID-19 vaccine doses across Australia [28]. More recently, the role of pharmacists has continued to evolve, with increasing involvement in routine and funded vaccination programs, further embedding pharmacies within the broader immunisation landscape [28, 29].

Evidence indicates that this expansion has been associated with improved vaccination accessibility, particularly through increased convenience, extended service times, and reduced need for appointments [30]. However, despite this expansion, there remains limited empirical evidence describing how pharmacist-administered vaccination services are utilised over time and across population groups and geographical areas in Australia. Existing studies have largely focused on scope expansion or policy impacts [21, 22, 28, 31], impact on vaccine uptake in the general population [25, 32], and have reported aggregated national immunisation coverage data [25, 27]. There has been less attention given to comprehensive analyses of temporal trends, demographic patterns, and geographic variation within pharmacy-based vaccination services.

To the best of our knowledge, limited studies have examined longitudinal, real-world data from community pharmacies to explore vaccination trends and patterns across demographic and geographic groups in Australia. Analysing such data offers a valuable opportunity to better understand service utilisation, access, and the role of pharmacists within the broader vaccination system and to identify areas of variation in service delivery.

### Aim

This study addressed the following research question: What are the temporal trends and demographic and geographic patterns of pharmacist-administered vaccinations within a predominantly Western Australian community pharmacy group? Specifically, it examined (i) trends in vaccination over time, (ii) variation patterns by age and sex, and (iii) differences in service distribution across Modified Monash Model (MMM) category and proximity to general medical centres.

## Method

### Study design and setting

This study employed a cross-sectional retrospective descriptive analysis of vaccination data from a predominantly Western Australian community pharmacy group. The selected pharmacy group is a major organisation [33] with established service delivery models, including pharmacist-administered vaccination programs [34]. It operates 86 community pharmacies across five Australian states, with 70 in Western Australia (WA), 10 in Victoria, and six in other states. We chose this pharmacy group based on its reach across metropolitan, regional, rural and remote locations, its professional service-focused operating model, and availability of routinely collected data over periods that align with the study objectives. This study was conducted from April to July 2025. Reporting for this study has been conducted using the STOBE checklist for observational studies (Supplementary file 1).

### Vaccination records and sample size

The study did not include active sampling and used a retrospective analysis of routinely collected data. The study included all vaccine administration data collected from community pharmacies in the selected pharmacy group between 1 January 2021 and 2 April 2025. Records with incomplete information on vaccine type and/or client demographics, as well as vaccination records used for system testing, were excluded from analysis.

### Variables

This study included vaccination temporal trends and vaccination patterns across demographic groups (age and sex) and geographical locations (MMM regions and proximity to medical centres) as the main outcomes. Age groups were categorised as younger (children and adolescents (aged 5–19 years), young adults (20–39 years), adults (40–64 years), older adults (65–84 years), and elderly (≥ 85 years). Sex group was categorised as male and female. Pharmacist geographic location was based on a postcode for the specific community pharmacy and was categorised based on MMM regions [35], which account for location remoteness and population distribution. Proximity to the medical centre providing vaccination services refers to the distance from each pharmacy to the nearest other healthcare setting providing vaccinations. It was determined using Google Maps, and it was classified into four groups based on median distance in kilometres (km).

In this study, the term “medical centre” refers to a relatively larger healthcare facility that includes multiple general practitioners alongside other healthcare services within the Australian context. We used “medical centre” consistently as an umbrella term when assessing vaccination proximity in relation to other vaccination providers, including general practices or GP clinics. In Australia, the terms “general practice” and “GP clinic” are sometimes used interchangeably.

### Data sources

The data sources were two consolidated administrative systems incorporating routinely collected vaccination data: GoBookings, a booking system (January 2021 to March 2024) and MedAdvisor, a clinical recording system (April 2024 to April 2025). Both data sources included vaccination appointment and service records from community pharmacies in the selected pharmacy group.

### Data cleaning and extraction

Data cleaning and management were conducted using Microsoft Excel 2019. Two authors (AKS and AT) cleaned and arranged the dataset from both sources before analysis. The initial routine data were anonymised and coded by AT, followed by data cleaning and organisation by AKS for subsequent analysis. Coding was independently reviewed to ensure clarity and consistency. The extracted variables include vaccination service or booking dates (including year and month), vaccine type, client age, and sex.

Data cleaning procedures included checking for duplicate records, identifying missing data entries, verifying variable completeness, and removing records that could not be reliably included in the analysis (for example, recording system testing bookings). The cleaned data was then organised into a structured format for descriptive analysis of vaccination service patterns over the study period.

### Data management and analysis

Vaccination trends were analysed by vaccine type and aggregated by vaccination year and month. Records for April 2025 were incomplete, so they were excluded from further trend analysis. Vaccination trends were illustrated by three groups considering the number of vaccinations counts for better visualisation of each vaccine: high-volume vaccines (COVID-19 and influenza), moderate-volume vaccines (dTpa and herpes zoster), and low-volume vaccines (cholera, dTpa/IPV, hepatitis A, hepatitis B, HPV, Japanese encephalitis, meningococcal, MMR, pneumococcal, poliomyelitis, rabies, RSV, typhoid, and varicella).

To present vacation patterns across demographic groups and geographic locations, vaccines were categorised into three groups based on vaccination counts: 1) COVID-19, 2) influenza, and 3) other vaccines (including cholera, diphtheria-tetanus-pertussis (dTpa), hepatitis A, hepatitis B, herpes zoster, human papilloma virus (HPV), Japanese encephalitis, meningococcal, measles-mumps-rubella (MMR), pneumococcal, poliomyelitis, rabies, respiratory syncytial virus (RSV), typhoid, and varicella). Vaccination patterns for COVID-19, influenza, and other vaccines therefore were separately analysed by age, sex, MMM classification, and proximity to the nearest medical centre.

Vaccination rates were calculated across demographic (age and sex) and MMM groups using records from the Western Australian subset of the pharmacy group, with other states excluded due to low representation and limited generalizability because of the limited number of pharmacies in the pharmacy group outside Western Australia. Rates were expressed per 100,000 population to enable standardised comparisons across groups with differing population sizes, consistent with epidemiological methods and reporting standards [36, 37].

For 2021–2024, rates were derived using aggregated vaccination counts within each subgroup as the numerator and cumulative population estimates of that subgroup (sum of annual estimates) as the denominator, stratified by age, sex, and MMM category using WA’s population demographic and geographic estimates [38, 39]. Rates were calculated as: (total vaccinations ÷ cumulative population) × 100,000. Influenza vaccination rates by age group across years were calculated annually using vaccination counts divided by the corresponding average annual population × 100,000 (Supplementary file 2).

Descriptive statistics, including frequencies and percentages, were used to summarise the data and were presented using line and bar graphs. Vaccination rates were compared across demographic and geographic groups. Associations between vaccine type and demographic or geographic categories were examined using the Pearson chi-square (χ^2^) test, with degrees of freedom (df) reported using IBM SPSS Statistics version 29. A two-sided *p*-value of < 0.05 was considered statistically significant.

### Ethics approval

Ethical approval was obtained from the Human Research Ethics Committee of Curtin University (approval number: HRE2025-0194). Participant consent was not required, and permission to access the data was received from the Pharmacy Group because this study relied on secondary data sources and no active sampling was required.

## Results

### Characteristics of vaccination records

**Fig. 1:**
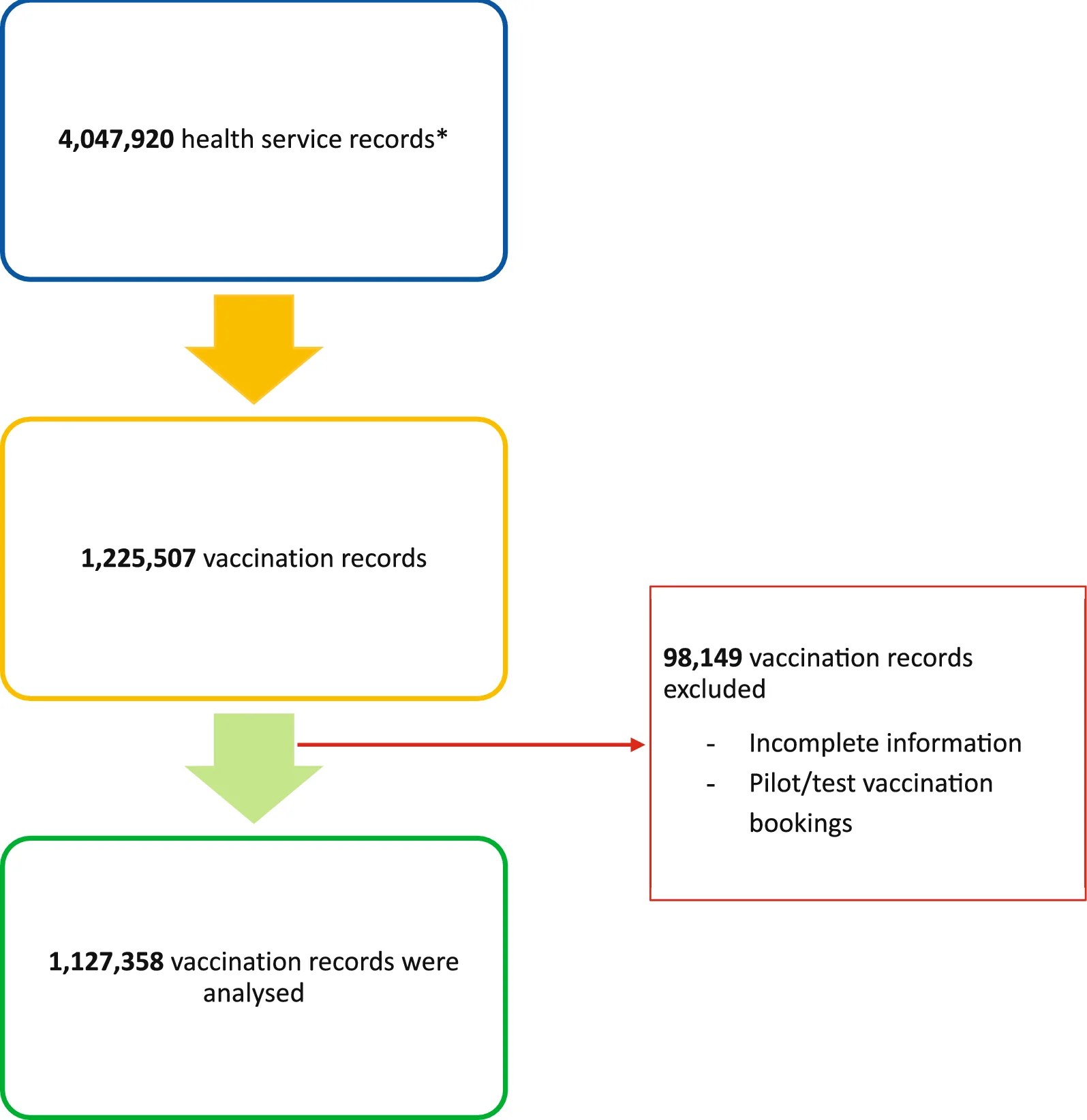
Inclusion of vaccination data from Pharmacy 777 Group pharmacies from 1 January 2021 to 2 April 2025. *Vaccination, screening (health checks), testing and monitoring, medication reviews, minor ailment management, chronic disease management, health promotion, consultations, weight loss programs

Between 1 January 2021 and 2 April 2025, 4,047,920 health service records showed that a total of 1,225,507 vaccinations were administered by the selected pharmacy group pharmacies in WA, Victoria (Vic), South Australia (SA), New South Wales (NSW), and Queensland (Qld). Of these, 1,127,358 ( 92%) records were included in the analysis, with the remainder excluded due to incomplete data and sample records used during system development(Fig. 1). Vaccination services were delivered across 85 community pharmacies, the majority of which are situated in WA, accounting for 99.2% of all recorded vaccinations. Approximately 90% of vaccinations were provided in pharmacies located in metropolitan (MM1) areas. Of the total vaccination records analysed, COVID-19 vaccines accounted for 59.4%, followed by influenza vaccines (37.8%) (Table 1).

**Table 1:** Characteristics of vaccination records (N = 1,127,358)

Variables		Vaccination counts	Percent (%)
Number of pharmacies			
WA	70	1,117,963	99.2
Vic	10	3,555	0.3
SA	2	3,172	0.3
NSW	2	2,057	0.2
Qld	1	611	0.05
Age group of clients			
5–19: younger individuals		142,167	12.6
20–39: young adults		310,645	27.6
40–64: adults		451,370	40.0
65–84: older adults		205,178	18.2
≥85: older population		17,998	1.6
Sex of clients			
Male		548,480	48.7
Female		554,534	49.2
Unspecified		24,344	2.2
Geographical location of vaccination provider pharmacies			
Metropolitan (MM1)		1,017,320	90.2
Regional (MM2)		40,953	3.6
Large rural (MM3)		33,494	2.9
Medium rural (MM4)		11,483	1.0
Small rural (MM5)		13,026	1.2
Remote/very remote (MM6-7)		11,082	1.0
Vaccine types delivered by pharmacists			
COVID-19		670,329	59.4
Influenza vaccines		425,767	37.8
Other vaccines*		31,262	2.8

*N* = *total vaccination counts;* WA = Western Australia, Vic = Victoria, SA = South Australia, NSW = New South Wales, Qld = Queensland; Other vaccines*: cholera, dTpa, hepatitis A, hepatitis B, herpes zoster, human papillomavirus (HPV), Japanese encephalitis, meningococcal, MMR, pneumococcal, poliomyelitis, rabies, RSV, typhoid, and varicella

### Vaccination trends

**Fig. 2:**
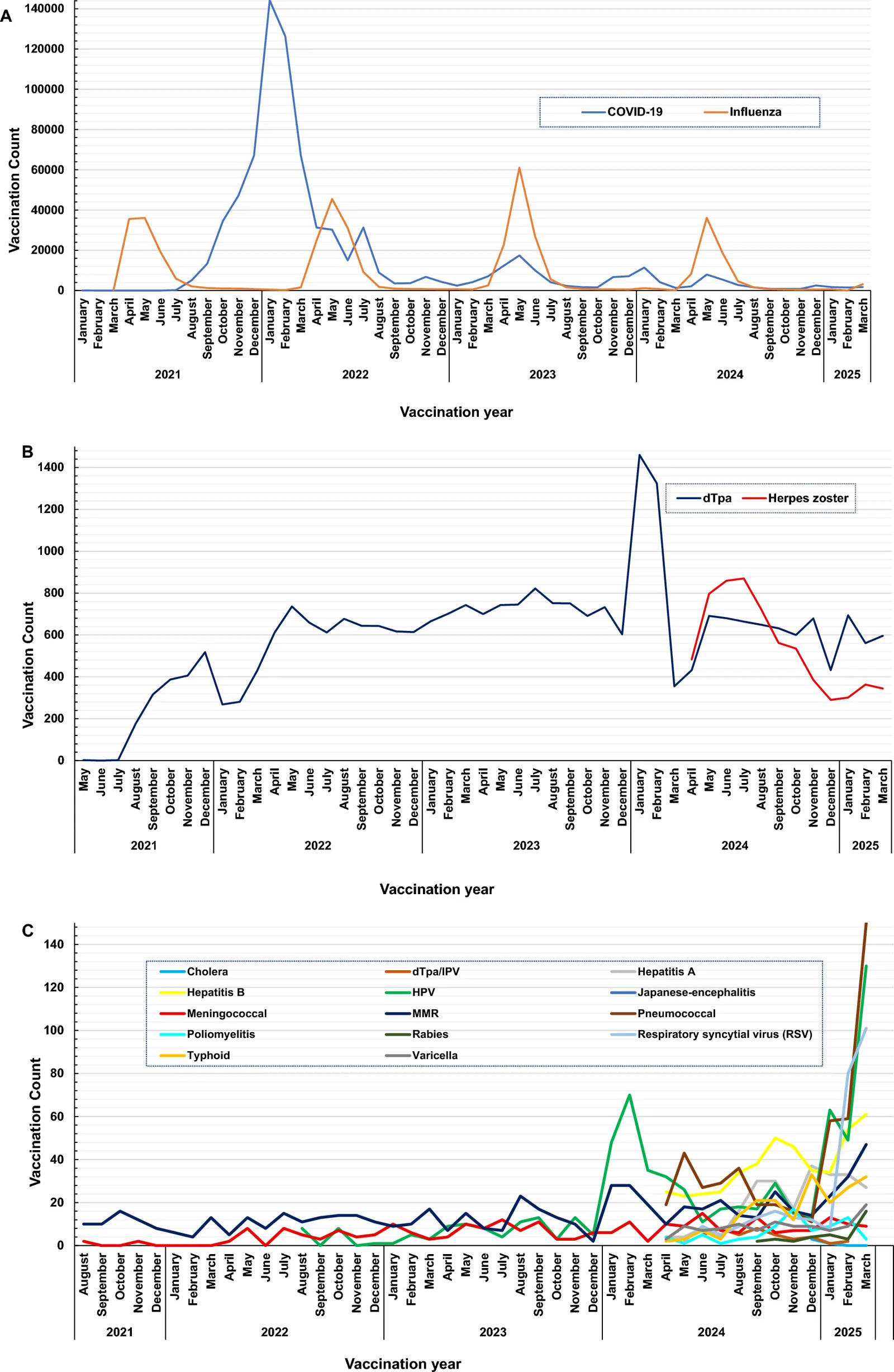
Vaccination trends by vaccine type from January 2021 to March 2025: **A** high-volume vaccines, **B** moderate-volume vaccines, **C** low-volume vaccines

From January to May 2021, the only vaccines administered by pharmacists were COVID-19, dTpa, and influenza. From August 2021, administration of meningococcal and MMR vaccines was recorded, followed by the HPV vaccine in August 2022. From April 2024 onwards, the range of vaccines administered significantly expanded to include cholera, dTpa/IPV, hepatitis A, hepatitis B, herpes zoster, Japanese encephalitis, pneumococcal, poliomyelitis, rabies, RSV, typhoid, and varicella (Fig. 2).

Seasonal variations in vaccination were observed across different vaccine types. COVID-19 vaccinations peaked between November 2021 and February 2022, accounting for approximately 475,000 administered doses during this period. A gradual decline followed, with a marked reduction from March 2023 and almost no COVID-19 vaccinations recorded from July 2024 to the end of the study period. In contrast, influenza vaccinations displayed a clear seasonal pattern, with peaks consistently occurring between March and June each year, and lower activity observed from July to February across the study period (Fig. 2A).

The dTpa vaccinations showed an initial upward trend from their introduction in July 2021 until May 2022, followed by a relatively stable pattern across subsequent seasons, with a notable peak between January and February 2023. In contrast, herpes zoster vaccinations increased between April and July 2024, but showed a steadily decreasing trend until December 2024 and then continued similarly through to the end of the study period (Fig. 2B).

Meningococcal, MMR, and HPV vaccines displayed similar trends following their introduction, although MMR and HPV showed a noticeable increase beginning in December 2023. Other vaccines, including pneumococcal, RSV, hepatitis A, hepatitis B, and typhoid, showed a slight upward trend from their commencement in April 2024. In contrast, vaccines such as cholera, dTpa/IPV, Japanese encephalitis, poliomyelitis, rabies, and varicella did not exhibit any noticeable increase in vaccinations following their introduction in April 2024 (Fig. 2C).

### Vaccination distribution

Vaccination patterns across age groups, sex, and MMM locations were estimated only for WA vaccination records, as vaccination numbers in other states were significantly lower, representing only 0.8% across all records, and did not reflect actual pharmacist-administered vaccinations in these states. However, vaccination distribution relating to medical centre proximity was calculated from the total vaccinations, including other states and the 2025 records, as these findings were not population dependent. Total vaccinations administered in WA from 2021 to 2024 numbered 1,112,302, with most being COVID-19 vaccinations (662,960; 59.6%), followed by influenza (420,070; 37.8%) and other vaccines (29,272; 2.6%) (Table 2).

**Table 2:** Vaccination counts by age group, sex, MMM category, and proximity to medical centre and cumulative WA population by age group, sex, MMM category (2021–2024)

Variables	Vaccination counts				
	Category	COVID-19	Influenza	Others	Total
Age group (years)	5–19	81,802	57,957	668	140,427
	20–39	185,755	106,862	13,590	306,207
	40–64	246,478	191,447	7,593	445,518
	65–84	135,794	59,846	6,757	202,397
	≥85	13,131	3,958	664	17,753
	Total	662,960	420,070	29,272	1,112,302
Sex	Male	323,831	205,192	12,243	541,266
	Female	327,613	206,931	12,680	547,224
	Unspecified	11,516	7,947	4,349	23,812
	Total	662,960	420,070	29,272	1,112,302
MMM category	MM1	604,266	373,705	25,809	1,003,780
	MM2	17,113	22,228	1,050	40,391
	MM3	17,307	14,487	1,228	33,022
	MM4	6,183	4,767	374	11,324
	MM5	8,961	3,355	528	12,844
	MM6-7	9,130	1,528	283	10,941
	Total	662, 960	420,070	29,272	1,112,302
Proximity to medical centre clinic (km)	< 0.6	367,700	301,167	19,496	688,363
	0.6–1.1	136,403	43,508	3,679	183,590
	1.2–1.7	137,120	64,104	6,954	208,178
	1.8–2.5	29,106	16,988	1,133	47,227
	Total	670,329	425,767	31,262	1,127,358

	Cumulative population estimates	
	Category	Four-year total population
Age group (years)	5–19	2,168,352
	20–39	3,299,487
	40–64	3,616,246
	65–84	1,601,174
	≥85	209,347
Sex	Male	5,327,049
	Female	5,391,360
MMM category	MM1	7,597,174
	MM2	472,635
	MM3	575,993
	MM4	248,278
	MM5	374,467
	MM6–7	373,488

### Age group

**Fig. 3:**
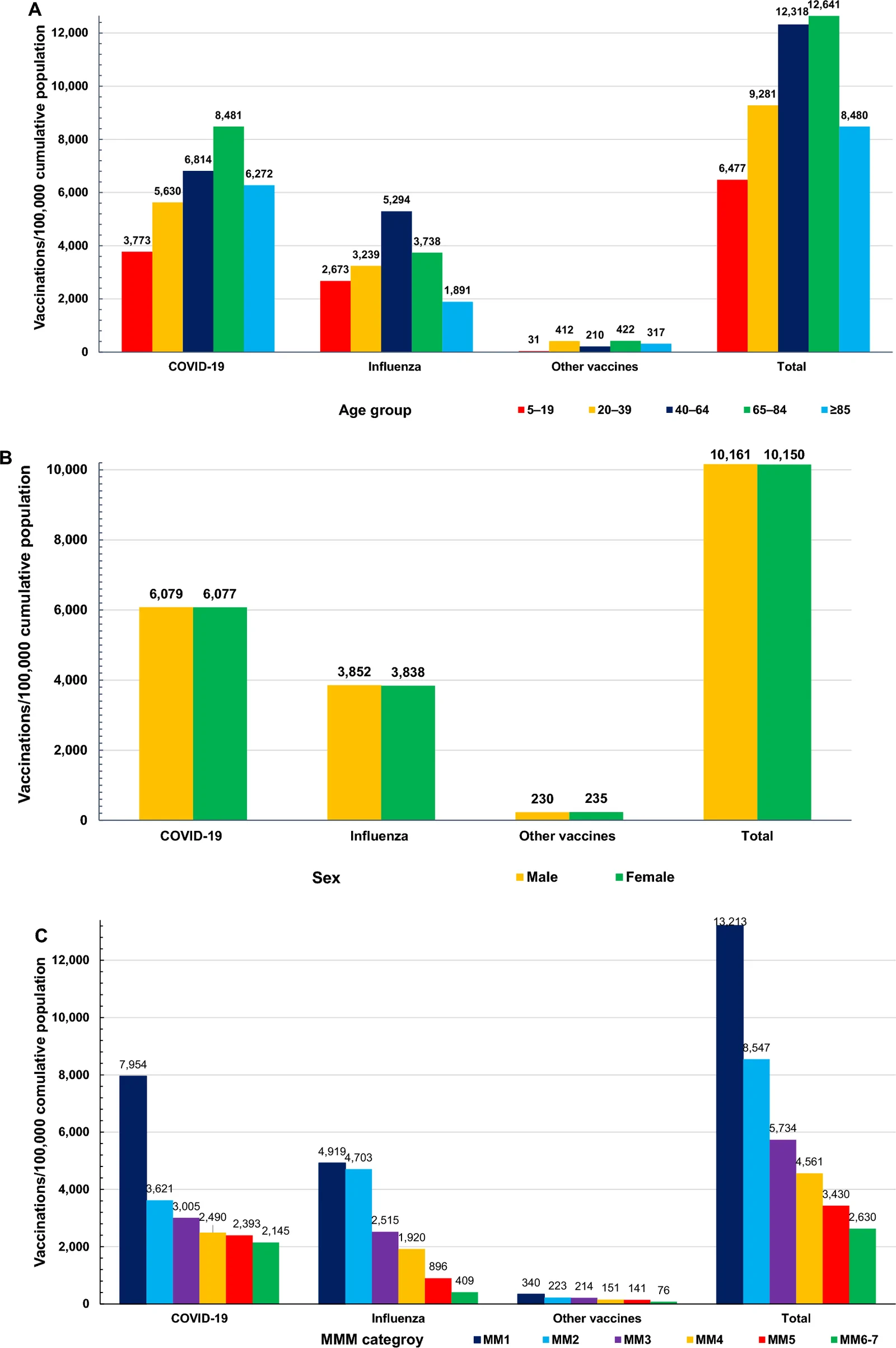
Vaccination distribution by demographic and geographic characteristics per 100,000 cumulative population: **A** by age group, **B** by sex, **C** by MMM category

The findings showed a statistically significant association between age group and vaccination by vaccine types (χ^2^(8) = 1,448, *p*<0.001), indicating different vaccination patterns between age groups. The overall vaccination rate was lower among younger individuals (aged 5–19 years) and elderly people (≥ 85 years) than that of other age groups, with rates of 6,477 and 8,480 vaccinations per 100,000 cumulative population, respectively. COVID-19 vaccination rates increased with age, peaking in the 65–84-year group before declining in those aged ≥85 years. Influenza vaccination showed a notable peak in the 40–64-year age group. For the other vaccines, although overall rates were considerably lower, a bimodal distribution was observed, with peaks in the 20–39 and 65–84-year age groups (Fig. 3A).

### Sex

The analysis revealed that there was no statistically significant association between sex and vaccination among vaccine types (χ^2^(2) = 0.2, *p* = 0.920), indicating that the distribution of COVID-19, influenza, and other vaccines was similar between males and females (Fig. 3B).

### Geographical regions

A statistically significant association was observed between the MMM category and vaccination by vaccine types (χ^2^(10) = 1,872, *p*<0.001), indicating differences in vaccination patterns across MMM categories and vaccine types. Overall, vaccination was relatively higher in metropolitan (MM1) areas, and vaccination rates decreased with increasing remoteness across all vaccines. COVID-19 vaccination was significantly higher in MM1 and declined from MM2 onwards, with relatively similar patterns observed across MM4–MM7. Influenza vaccination rates were comparable in MM1 and MM2, followed by a decrease in MM3 and MM4 (which showed relatively similar patterns), with a further significant decline in MM5–MM7. Vaccinations for other vaccines demonstrated a gradual decrease from MM1 through MM6/7 (Fig. 3C).

### Proximity to a medical centre

**Fig. 4:**
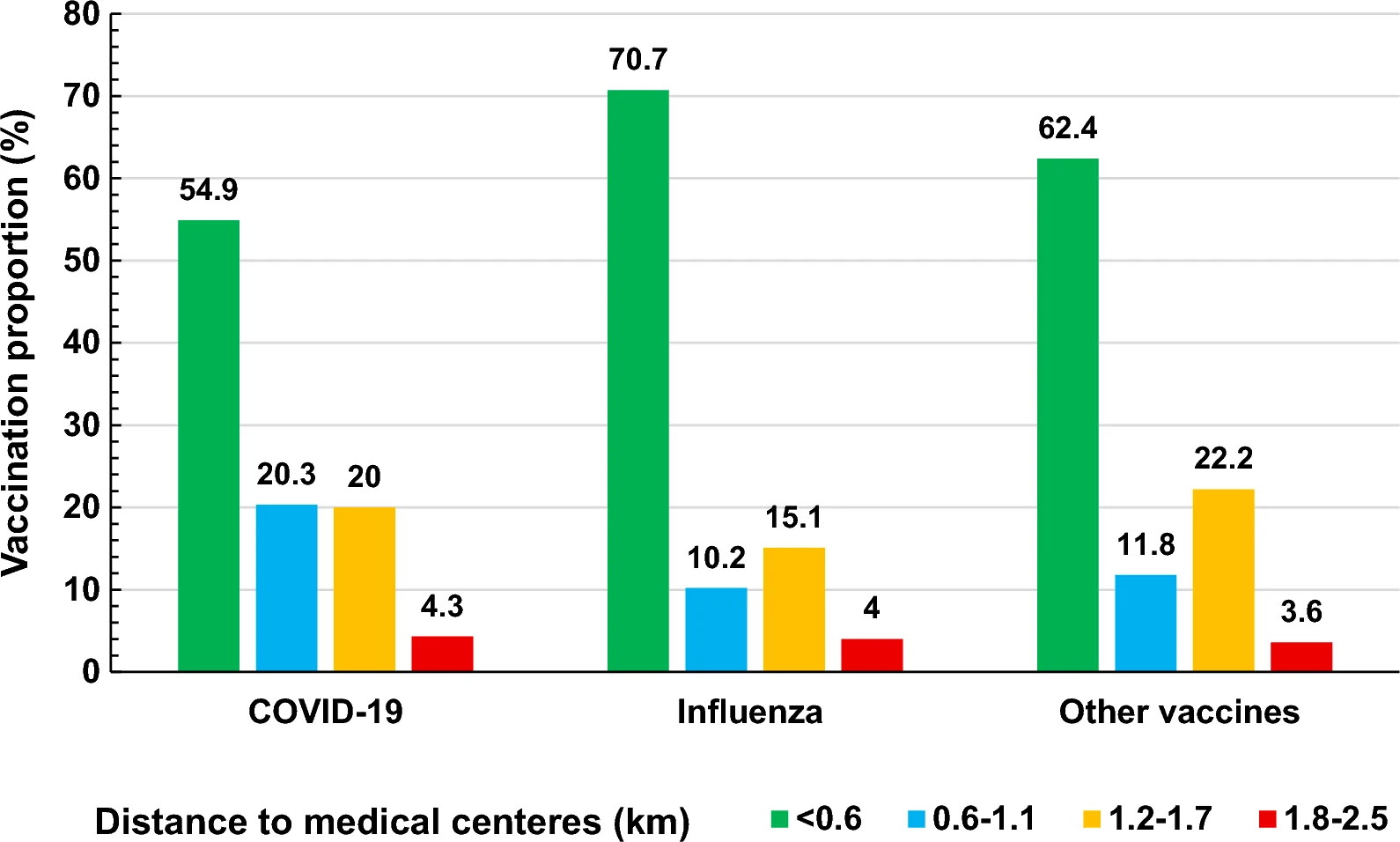
Vaccination distribution by proximity to medical centre

Most pharmacist-administered vaccines were administered in pharmacies within 0.6 km of a medical centre: 70.7% of influenza, 62.4% of other vaccines, and 54.9% of COVID-19 vaccinations (Fig. 4).

### Influenza vaccination distribution by age across years

**Fig. 5:**
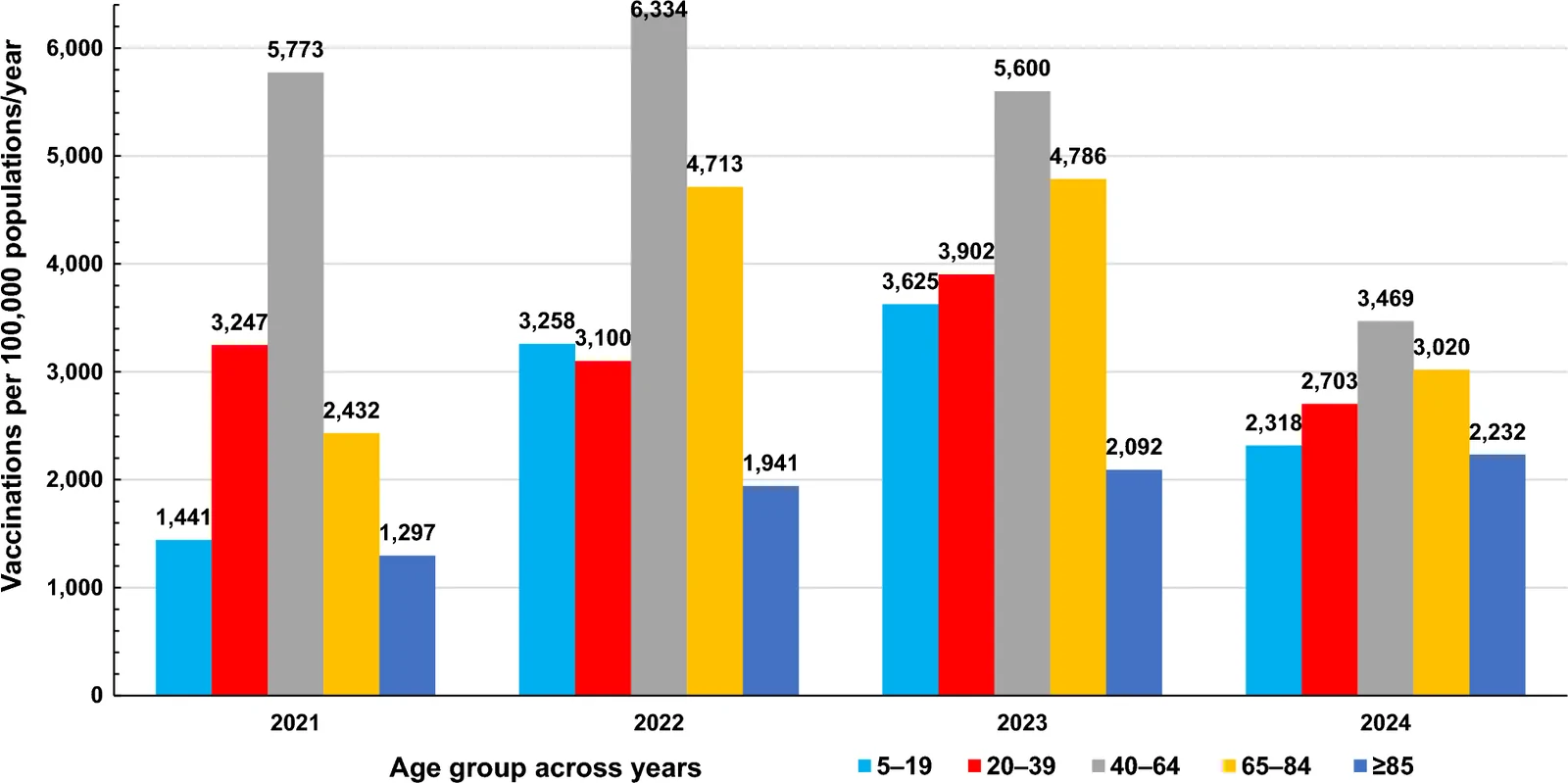
Influenza vaccination distribution per 100,000 population per year by age group from 2021 to 2024

Influenza vaccination patterns across years differed significantly by age group over time (Table 3), with a strong association between age group and year (χ^2^(12) = 1849, *p* < 0.001). Vaccination peaked in 2022 and 2023 across most age groups. Notably, vaccination rates declined across all age groups in 2024 compared to 2022 and 2023. However, the ≥85 years age group demonstrated a distinct pattern, with the rate increasing in 2024 (Fig. 5).

**Table 3:** Influenza vaccination by age group across years and WA four-year average annual population [2021–2024]

Age group	Vaccination counts by year				
	2021	2022	2023	2024	Total
5–19	7,849	17,745	19,742	12,621	57,957
20–39	26,793	25,576	32,193	22,300	106,862
40–64	52,188	57,267	50,631	31,361	191,447
65–84	9,735	18,866	19,156	12,089	59,846
≥85	679	1,016	1,095	1,168	3,958
Total	97,244	120,470	122,817	79,539	420,070
	Four-year average annual population estimates				
5–19	544,588				
20–39	825,122				
40–64	904,062				
65–84	400,294				
≥85	52,337

## Discussion

To the best of our knowledge, this is the first study providing a detailed examination of temporal trends and population-level patterns in pharmacist-administered vaccinations within a predominantly Western Australian community pharmacy group. The findings demonstrate that vaccination activity in the study setting is not static but has evolved in response to broader public health context, population needs, and service accessibility. Over the study period, vaccination delivery transitioned from a concentrated focus on COVID-19 toward a more diverse portfolio of vaccines, while also revealing consistent patterns across age groups and geographic regions, with concentration in metropolitan centres and pharmacies located near medical centres. These patterns highlight the complexity of vaccination service utilisation in community pharmacy settings and the interplay between demand, accessibility, and existing healthcare structure.

The temporal trends observed reflect a clear shift in the role of community pharmacies over time. The initial dominance of COVID-19 vaccinations corresponds to a period of rapid scale-up in vaccination delivery, where pharmacists became key points during a high -demand phase. This pattern is consistent with national [28, 40] and international observations [41, 42], where pharmacies were integrated into emergency vaccination efforts to increase coverage and accessibility. The subsequent decline in COVID-19 vaccination activity likely reflects a combination of reduced perceived risk, removal of vaccination mandates, changing eligibility criteria, and growing vaccine fatigue. Similar patterns globally have shown a broader shift in vaccination behaviours as countries move beyond the acute phase of the pandemic [43–45].

Seasonal influenza vaccination trends provide further insights into how pharmacists' role aligns with established immunisation cycles. These patterns are consistent with evidence from other settings in high-income countries [46, 47]. The consistent annual peaks align closely with national vaccination campaigns, suggesting that pharmacists are well positioned to support immunisation efforts during periods of high demand by leveraging their accessibility and convenience [20, 30]. However, fluctuations in the magnitude of these peaks over time, for instance relatively lower peaks in 2024, may indicate changing public perceptions of risk and vaccine fatigue, particularly in the post-pandemic context. This finding aligns with an Australian report that showed a post-pandemic plateau trend [48]. A study from the United States (US) also showed a decrease in influenza vaccination patterns compared to previous years [49]. These patterns may suggest that while pharmacists effectively support campaign-based vaccination delivery, uptake remains sensitive to external factors such as public awareness and perceived need, underscoring the need for sustained public health engagement and strategies to maintain vaccination momentum beyond periods of heightened awareness.

Beyond COVID-19 and influenza, the delivery of a broader range of vaccines by community pharmacists reflects a continued expansion of vaccination services within community pharmacy settings. Recent Australian Immunisation Register (AIR) data indicate a gradual expansion in pharmacist-administered vaccination, with an increasing proportion of vaccinations delivered by pharmacists following our study period, particularly between 2024 and June 2026 [50]. This expansion is particularly evident in vaccines such as herpes zoster and pneumococcal vaccines, as well as in the delivery of respiratory vaccines, including COVID-19, influenza, and respiratory syncytial virus (RSV). Although trends vary across vaccine types over time [50], the data suggest this expansion is supporting increased vaccine coverage rather than solely representing a shift in vaccine provision. It also likely reflects growing public acceptance of pharmacies as vaccination providers, alongside increasing familiarity with pharmacist-administered vaccinations, with many people choosing pharmacies for their accessibility and convenience [30]. However, variation across vaccine types suggests that uptake is not uniform and may be influenced by vaccine-specific factors such as eligibility criteria, cost, awareness, provider preferences, and perceived importance [51, 52]. Temporal peaks observed in certain vaccine types, including dTpa, may correspond to episodic demand driven by travel, occupational requirements, or targeted health campaigns [53]. In contrast, stable or limited uptake for other vaccines, for instance, herpes zoster, indicates structural (e.g., funding arrangements, supply limitations, and limited prescription authorisation to physicians) or behavioural barriers may persist, limiting broader utilisation despite service availability [54]. In addition, vaccines with more restricted indications, such as travel-related or niche vaccines, are not administered in WA under the Structured Administration and Supply Arrangement (SASA) framework, continue to account for a small proportion of overall activity, a pattern consistent with international evidence [55, 56].

The observed expansion in the range of routine childhood vaccines in the selected community pharmacies since January 2024 indicates a shift toward broader service provision, aligned with legislative changes and national workforce strategies [29, 57]. However, variability in uptake across vaccine types suggests that policy, regulation, and public awareness remain key determinants of service utilisation. Notably, uptake of certain vaccines did not demonstrate a meaningful increase following their introduction. This may reflect jurisdiction limitations, as several vaccines were not permitted for administration in WA under the SASA framework. Consequently, these findings likely represent activities from a limited number of pharmacies within the selected group operating in other states and territories [58].

Demographic patterns observed in this study reveal important differences in how vaccination services are accessed across age groups. The overall lower uptake among younger (aged <19 years) and older populations (aged ≥85 years) likely relates to structural and behavioural factors influencing vaccination utilisation. However, a study by Isenor et al. [59] showed that pharmacists' involvement increases overall vaccination uptake in children and older populations rather than comparing uptake among population groups. School-based immunisation programs [60] and existing care pathways (e.g., WA’s jurisdictional restrictions for most vaccines) may further limit pharmacist-based services engagement for younger populations [58].

The relatively lower uptake among older populations may suggest more complex patterns of healthcare engagement, potentially influenced by mobility limitations, higher levels of comorbidity, established relationships with traditional vaccinators, funding pathways, and the presence of aged care homes [61, 62]. The exceptionally high COVID-19 vaccination rates observed among older adults and older people may reflect the disproportionate burden of COVID-19 morbidity and mortality in these groups [63]. Older adults were prioritised in vaccination programs and were more likely to seek vaccinations through accessible healthcare settings, including community pharmacies [64]. The increased vaccination rate among individuals aged ≥85 years in 2024 may reflect improved access following the introduction of the NIPVIP program, which expanded funded pharmacist-administered vaccination services from January 2024 [21, 28, 29]. The AIR data also showed an increased vaccination pattern among this population in 2024 compared to a previous year [50]. However, the observed pattern should be interpreted cautiously, as changes in service availability, reporting practices, and other contextual factors may have influenced vaccination uptake. In contrast, higher vaccination utilisation among the adult population may also reflect the alignment of pharmacy services with adult vaccination needs, particularly in terms of accessibility and convenience, including flexible hours and walk-in availability. These patterns are consistent with Australian and international evidence, where pharmacists are more commonly utilised by working-age adults who value convenience, accessibility, and autonomy in healthcare decisions [61].

The minimal differences observed between males and females suggest a relatively uniform pattern of pharmacy-based vaccination across sex groups. This contrasts with some previous evidence indicating that preventive health behaviours can vary by sex, with females often demonstrating high engagement [65], particularly for specific vaccines such as HPV [66, 67]. This absence of substantial differences in this study may reflect the accessibility and convenience of pharmacy-based services, which reduce traditional barriers to care such as appointment requirements and time constraints. During periods of heightened vaccination demand, such as the COVID-19 pandemic, widespread public messaging and vaccination mandates may have further contributed to similar uptake among these population groups. These findings suggest that community pharmacy settings may support more equitable access to vaccination services in terms of sex-based utilisation.

Geographical variation provides additional context for understanding how vaccination services are distributed and accessed. The higher concentration of vaccinations in metropolitan areas, with relatively lower rates across regional and rural regions, is likely driven by the distribution of the participating pharmacies within the areas, which may reflect differences in service availability, workforce distribution, and local healthcare infrastructure [68]. Although pharmacies are often positioned as accessible healthcare providers, including in rural areas [69], their reach and impact may still vary substantially across geographic contexts, and it also raises the possibility of “vaccine deserts” [70, 71], where access to vaccination providers is limited in the current case. In addition, socioeconomic and sociocultural variations across geographical regions may further influence vaccination uptake [72]. Such gaps may occur not only in rural settings but also within underserved urban areas. Limitations in workforces, including availability of vaccinator pharmacists, infrastructure, and service availability, may constrain their impact. The proximity of pharmacy vaccination to other vaccination provider settings further suggests the importance of co-location. Interestingly, this challenges assumptions that pharmacies only fill gaps in areas with limited primary healthcare access [69]. Rather than a substitution or duplication of services, this co-location within medical precincts may reflect complementary service provisions, where pharmacies and other vaccinators collectively contribute to consumers' greater flexibility and meeting vaccination demand. Such patterns emphasise that pharmacy-based vaccination services can operate within a broader system of care, offering walk-in accessibility, longer hours, and shorter wait times [20, 30].

Overall, the trends and patterns observed in the included pharmacies in this study indicate that pharmacist-administered vaccination services are dynamic and responsive to changing population needs. Temporal variation reflects broader public health events and shifting demand, while demographic and geographic patterns highlight differences in access and utilisation across population groups. These findings underscore the importance of considering both population characteristics and service context when interpreting vaccination activity within community pharmacy settings.

### Implications for pharmacy practice and policy

The findings highlight the valuable role of community pharmacies in expanding vaccination access and coverage. Seasonal and demographic patterns underscore the need for targeted planning, workforce optimisation, and tailored outreach. Overall, coordinated action across practice and policy is essential to maximise the impact of pharmacy-based vaccination services and improve equity.

#### Practice implications

Strengthening data quality and service efficiency remains essential, including ongoing monitoring, routine audits, and staff training to ensure accurate recording and effective service delivery. Proactive service planning, such as aligning staffing and stock management with seasonal demand, can improve responsiveness during peak periods. Evaluating service distribution, particularly in rural and underserved areas and population groups, would help ensure more equitable access and maximise the impact of pharmacist-based vaccination programs. Targeted communication and outreach strategies are needed to address lower uptake in specific population groups and regions.

#### Policy implications

Expanding and standardising the scope of practices, along with consistent regulatory and funding models, could enhance system capacity, reduce pressure on other vaccinators, and strengthen preparedness for future public health demands [73]. Targeted investments in rural and regional pharmacy infrastructure and workforce would enhance uptake. The observed co-location with medical centres highlights the value of integrated models. Policies could support collaboration between pharmacies and other primary care providers to improve coordination, continuity of care, and overall vaccination coverage [74, 75].

### Strengths and limitations

This study provides comprehensive insights into vaccination patterns within a predominantly Westen Australian community pharmacy group, drawing on a larger dataset over four-year period. The extended timeframe enabled identification of temporal trends, including seasonal variations, fluctuations in uptake, and changes associated with evolving service delivery. The dataset also allowed examination of patterns across key demographic and geographic characteristics.

However, several limitations should be considered when interpreting the findings. The exclusion of incomplete records may have led to an underestimation of vaccination volumes and trends in some periods. As the data were drawn from a single pharmacy group, predominantly located in WA, the generalisability of the findings to the broader Australian context is limited. The relatively low proportion of vaccinations observed in regional and rural areas likely reflects the geographical distribution of participating pharmacies rather than true population-level uptake.

In addition, this study did not include vaccinations delivered by other providers or pharmacist-administered vaccinations outside the selected pharmacy group. While AIR data have recently become publicly available [50], these data were not available at the time of analysis and do not cover the full study period. Therefore, comparing our findings with this aggregated data set was outside the scope of this study, although it may provide useful context for future research by helping to determine the overall contribution of community pharmacies to vaccination coverage. Furthermore, because all vaccination records were included, repeat vaccinations for the same individual may have been counted; therefore, population-level patterns and rates should be interpreted with caution. Despite these limitations, this study provides real-world evidence on vaccination patterns over an extended period, capturing variation across time, population groups, and geographic settings within community pharmacy practice.

### Future studies

Further research is needed to better understand the drivers of lower community pharmacy vaccination rates in specific population groups and MMM categories, including preferences for vaccination settings and behavioural factors beyond eligibility and regulation. Future studies should examine broader determinants of uptake, including socioeconomic factors, access to services, and community-level influences using comprehensive national population data. Research into client attitudes and confidence in pharmacist-administered vaccination would support more tailored, client-centred service design. Additionally, exploring the impact of service co-location with other vaccinators could provide insights into how integrated models influence vaccination utilisation and access.

## Conclusion

Community pharmacists in the participating pharmacy group delivered a substantial volume of vaccinations over the study period, demonstrating their key role in immunisation efforts, particularly during the COVID-19 pandemic and seasonal influenza periods. While these findings highlight the accessibility and capacity of community pharmacists to support vaccination delivery, the extent to which these services represent additional uptake versus a shift from traditional providers requires further investigation. Nevertheless, the observed variations across demographic and geographic groups suggest opportunities for targeted strategies to improve vaccination access and coverage among under-vaccinated populations.

## Supplementary Information

Below is the link to the electronic supplementary material.

Supplementary file1 (DOC 84 KB)

Supplementary file2 (DOCX 34 KB)

## Data Availability

The de-identified datasets generated and/or analysed during the current study are available from the corresponding author upon reasonable request.
