# Supplementary material for: Trends and patterns of pharmacist-administered vaccination in a predominantly Western Australian pharmacy group: retrospective analysis of routinely collected data
Source: Int J Clin Pharm. 2026 Sep 15;48(6):2440–55. doi: 10.1007/s11096-026-02221-0 (PMC13731499; doi:10.1007/s11096-026-02221-0)
Supplement: Supplementary file 1 [file 11096_2026_2221_MOESM2_ESM.docx]

**Calculation of vaccination distributions for age, sex, and MMM category**

Vaccination distributions per 100,000 population were calculated for age group, sex, and MMM category using the total number of vaccination counts accumulated over the four years as the numerator and the corresponding cumulative population over the same four-year period as the denominator. The estimates were calculated using the following formula:

***Vaccinations per 100,000 population = (Total vaccination records for the subgroup/Cumulative population of the subgroup over four years) × 100,000****.*

For example, the rate of COVID-19 vaccination among individuals aged 5–19 years was calculated as 81,802 vaccination records divided by a cumulative population of 2,168,352, multiplied by 100,000, resulting in 3,773 vaccinations per 100,000 cumulative population.

Similarly, vaccination distributions by sex were calculated using the total vaccination records for each sex group divided by the corresponding four-year cumulative population. For example, the COVID-19 vaccination rate among males was calculated as 323,831/5,327,049 × 100,000, resulting in 6,079 vaccinations per 100,000 cumulative population.

Vaccination distributions by MMM category were calculated using the same approach. For example, the COVID-19 vaccination rate for MM1 areas was calculated as 604,266/7,597,174 × 100,000, resulting in 7,954 vaccinations per 100,000 cumulative population. The same calculation was applied to all other age, sex, and MMM categories, using the vaccination records and population estimates presented in Table 2, resulting in a vaccination rate presented in Figure 3 in the manuscript.

**Table 1.** WA Four-year population estimates by age, sex, and MMM category from 2021 to 2024 [1-5].

| Variables | Category | Four-year total population | 2021 | 2022 | 2023 | 2024 |
| --- | --- | --- | --- | --- | --- | --- |
| Age group (years) | 5–19 | **2,168,352** | 522,379 | 530,847 | 549,278 | 565,848 |
|  | 20–39 | **3,299,487** | 787,092 | 802,411 | 838,370 | 871,614 |
|  | 40–64 | **3,616,246** | 878,052 | 889,521 | 913,535 | 935,138 |
|  | 65–84 | **1,601,174** | 378,509 | 392,305 | 408,452 | 421,908 |
|  | ≥85 | **209,347** | 50,106 | 51,540 | 53,090 | 54,611 |
| Sex | Male | **5,327,049** | 1,283,343 | 1,304,147 | 1,349,425 | 1,390,134 |
|  | Female | **5,391,360** | 1,298,837 | 1,319,891 | 1,365,716 | 1,406,916 |
| MMM category | MM1 | **7,597,174** | 1,815,079 | 1,849,205 | 1,930,883 | 2,002,008 |
|  | MM2 | **472,635** | 114,932 | 116,726 | 119,375 | 121,601 |
|  | MM3 | **575,993** | 139,424 | 141,998 | 145,670 | 148,901 |
|  | MM4 | **248,278** | 60,278 | 61,330 | 62,682 | 63,988 |
|  | MM5 | **374,467** | 91,242 | 92,912 | 94,311 | 96,002 |
|  | MM6–7 | **373,488** | 92,131 | 92,773 | 93,127 | 95,457 |

*MMM: Monash Modified Model; population estimates are based on previous reports from the Australian Bureau of Statistics and are approximate estimates, not actual numbers; the population size of under five has been reduced in all variables.*

**Calculation of influenza vaccination distribution by age group across**

Annual vaccination rates per 100,000 population were calculated for each age group by dividing the number of vaccination records for the respective age group in each year by the corresponding average annual population for that age group and multiplying by 100,000.

Vaccinations per 100,000 population per year = (Vaccination records for the age group in a specific year/Average annual population of the age group) × 100,000.

For example, the COVID-19 vaccination rate among individuals aged 5–19 years in 2021 was calculated as 7,849 vaccination records divided by an average annual population of 544,588, multiplied by 100,000, resulting in 1,441 vaccinations per 100,000 population per year.

We applied the same calculation to all age groups and years using the vaccination counts and corresponding average annual population estimates presented in Table 3. The resulting annual vaccination rates are presented in Figure 5 of the manuscript.

**Table 2**. WA annual population estimates by age from 2021 to 2024 [1-3].

| Age group | Average annual population | 2021 | 2022 | 2023 | 2024 |
| --- | --- | --- | --- | --- | --- |
| 5–19 | **544,588** | 522,379 | 530,847 | 549,278 | 565,848 |
| 20–39 | **825,122** | 787,092 | 802,411 | 838,370 | 871,614 |
| 40–64 | **904,062** | 878,052 | 889,521 | 913,535 | 935,138 |
| 65–84 | **400,294** | 378,509 | 392,305 | 408,452 | 421,908 |
| ≥85 | **52,337** | 50,106 | 51,540 | 53,090 | 54,611 |

*Population estimates are based on previous reports from the Australian Bureau of Statistics, and they are approximate, not actual numbers.*

**Calculation of vaccination distribution by proximity to medical centres**

Proximity to medical centres was expressed as the percentage of vaccinations occurring within each distance category relative to the total number of vaccinations for each vaccine type. The percentage was calculated using the following formula:

Percentage of vaccinations within each distance category = (Vaccination records within the distance category / Total vaccination records for the respective vaccine type) × 100.

For example, 367,700 COVID-19 vaccinations occurred within <0.6 km of a GP clinic/medical centre, out of 670,329 total COVID-19 vaccinations. Thus, the percentage was calculated as 367,700 / 670,329 × 100 = 54.9%.

**Table 3.** Vaccination distributions by proximity to GP clinics/medical centres in all community pharmacies.

| Proximity to nearest GP clinic (km) | Type of vaccines (vaccination, %) | | |
| --- | --- | --- | --- |
|  | COVID-19 | Influenza | Others |
| <0.6 (n=688,363) | 367,700 (54.9) | 301,167 (70.7) | 19,496 (62.4) |
| 0.6-1.1 (n=183,590) | 136,403 (20.3) | 43,508 (10.2) | 3,679 (11.8) |
| 1.2-1.7 (n=208,178) | 137,120 (20.5) | 64,104 (15.1) | 6,954 (22.2) |
| 1.8-2.5 (n=47,227) | 29,106 (4.3) | 16,988 (4.0) | 1,133 (3.6) |
| Total (N=1,127,358) | **670,329 (59.4)** | **425,767 (37.8)** | **31,262 (2.8)** |

*.*

**References**

1. Australian Bureau of Statistics. Regional population by age and sex, 2024. Available at <https://www.abs.gov.au/statistics/people/population/regional-population-age-and-sex/latest-release>. Accessed on 20-05-2026.

2. Australian Bureau of Statistics. National, state and territory population. Available at <https://www.abs.gov.au/statistics/people/population/national-state-and-territory-population/jun-2024#2021-census-update-of-the-net-interstate-migration-model>. Accessed on 20-05-2026.

3. Australian Bureau of Statistics. 2021 Census All persons QuickStats (Western Australia). Available at <https://www.abs.gov.au/census/find-census-data/quickstats/2021/5>. Accessed on 20-05-2026

4. Australian Government. Department of Health. Modified Monash Model. Available at <https://www.health.gov.au/sites/default/files/2025-07/modified-monash-model-fact-sheet.pdf>. Accessed on 20-05-2026.

5. Australian Department of Health, Disability and Ageing. Modified Monash Model. Available at

<https://www.health.gov.au/topics/rural-health-workforce/classifications/mmm>. Accessed on 20-05-2026.
